# Hyperprolactinemia with Galactorrhea Due to Subclinical Hypothyroidism: A Case Report and Review of Literature

**DOI:** 10.7759/cureus.2723

**Published:** 2018-05-31

**Authors:** Kashif Aziz, Amir Shahbaz, Muhammad Umair, Mohaddeseh Sharifzadeh, Issac Sachmechi

**Affiliations:** 1 Internal Medicine, Icahn School of Medicine at Mount Sinai Queen Hospital Center, New York, USA

**Keywords:** primary hypothyroidism, subclinical hypothyroidism, levothyroxine, hyperprolactinemia, galactorrhea

## Abstract

Hyperprolactinemia is a common finding in primary hypothyroidism, but increased prolactin in the setting of subclinical hypothyroidism (SCH) has been scarcely reported in the literature. This is a rare case of hyperprolactinemia due to SCH that resolved with thyroid hormone replacement therapy. The patient was not on any medications known to cause hyperprolactinemia but she was using isoniazid for her latent tuberculosis. Isoniazid therapy may explain breast pain, but there is no reported relationship between isoniazid use causing subclinical hypothyroidism and hyperprolactinemia. A literature review reveals that few cases of galactorrhea associated with subclinical hypothyroidism have been reported. Similar to the reported cases in the literature, our patient’s thyroid stimulating hormone (TSH) and prolactin levels returned to normal with levothyroxine therapy.

## Introduction

Hyperprolactinemia is common in primary hypothyroidism, but, to our knowledge, marked elevation of serum prolactin in subclinical hypothyroidism (SCH) has not been commonly reported (Sachmechi I, Reich D, Bhatti H, Kim P: Hyperprolactinemia with galactorrhea due to subclinical hypothyroidism, abstract. 2010 Abstract, The American Association of Clinical Endocrinologists, (2010), https://www.aace.com/files/abstracts-2010.pdf ). Prolactin is a pituitary hormone that plays an important role in a number of reproductive functions. Hyperprolactinemia can result from many causes, including hypothyroidism and pituitary disorders as well as medication use. Depending on the cause and outcomes of hyperprolactinemia, selected patients need treatment [1]. Prolactin is under dual regulation by hypothalamic hormones delivered through the hypothalamic-pituitary circulation. The predominant signal is inhibitory, preventing prolactin release and is mediated by dopamine. The stimulatory signal is mediated by the hypothalamic hormone, thyrotropin-releasing hormone (TRH). In patients with hypothyroidism, an increase in prolactin is due to compensatory increase in the discharge of central hypothalamic TRH as a result of low thyroxine [1]. In the few hypothyroid patients who have elevated serum prolactin levels, the values become normal when the hypothyroidism is corrected [2]. Here we report a case of hyperprolactinemia due to SCH, in which the prolactin level became normal after levothyroxine treatment.

## Case presentation

A 48-year-old female from Honduras presented to the clinic with chief complaints of breast tenderness and galactorrhea for the past two to three weeks. She had a past medical history of hypertension, latent tuberculosis (TB) and sciatica. Her last menstrual period was three weeks prior to her presentation. Physical examination showed non-tender, diffuse enlargement of the thyroid gland, which was unchanged over the past one year. On palpation, breast examination revealed bilateral tenderness and milky yellowish discharge. The visual field testing and rest of the general physical examination were within normal limits. She was taking hydrochlorothiazide 25 mg and losartan 50 mg daily for hypertension and was on isoniazid (INH) and vitamin B6 for the treatment of latent tuberculosis. She denied the use of tobacco, marijuana, alcohol, illicit drugs, or over the counter medicines. Laboratory investigations are given below (Table 1).

**Table 1:** Laboratory Findings Abbrevations: Thyroid Stimulating Hormone (TSH), Free Thyroxine (FT4), Serum Throxine (Total T4), Total Triiodothyronine (Total T3)

Test	Units	Normal Range	Patient values
TSH	mIU/ml	0.7-5	5.63
FT4	ng/dl	0.58-1.64	0.75
Total T4	mcg/dl	6.09-12.2	6.96
Total T3	ng/dl	87-178	91.4
Prolactin	ng/ml	3.34-26.74	55.42

She had negative urine pregnancy test. Her mammogram was normal, and magnetic resonance imaging (MRI) of the brain didn’t show any pituitary mass. After looking at her thyroid function tests, prolactin level, and other respective tests, a diagnosis of subclinical hypothyroidism with hyperprolactinemia was made. She was prescribed levothyroxine 50 mcg daily and three months later her galactorrhea and breast tenderness were relieved. Her repeat blood testing showed normal thyroid stimulating hormone (TSH) level 1.4 mIU/ml and normal serum prolactin level of 13.44 ng/ml.

## Discussion

Prolactin is a peptide hormone produced by lactotroph cells of the anterior pituitary gland and is primarily associated with lactation. Hyperprolactinemia results almost exclusively from diseases that cause hypersecretion of prolactin by lactotroph cells. Physiologic causes include pregnancy, nipple stimulation, and stress. Pathologic causes include lactotroph adenomas, other hypothalamic and pituitary disorders. It can be caused by medications, most commonly antipsychotics, antidepressants, and antiemetics [2]. Other causes include idiopathic hyperprolactinemia, hypothyroidism, estrogen administration, chronic renal failure, and macroprolactinemia. A germline, loss-of-function mutation in the prolactin receptor gene (PRLR) resulting in prolactin insensitivity has been identified as a cause of familial hyperprolactinemia [2]. In the United States, this condition occurs in less than 1% of the general population and in 5-14% of patients presenting with secondary  amenorrhea. Approximately 75% of patients presenting with galactorrhea and amenorrhea have hyperprolactinemia. Of these patients, approximately 30% have prolactin-secreting tumors [3]. On review of literature, we found one case report of a patient with SCH associated with hyperprolactinemia that normalized with levothyroxine therapy and another case report regarding isoniazid-associated, painful, bilateral gynecomastia [4]. In our case the painful gynecomastia could be related to isoniazid use, but the galactorrhea is due to hyperprolactinemia. Since the treatment of her SCH with levothyroxine resolved her hyperprolactinemia and galactorrhea along with normalization of her TSH, it concludes that the hyperprolactinemia was due to SCH. Review of literature regarding hyperprolactinemia and its association with SCH is given below (Table 2).

**Table 2:** Review of Literature Abbrevations: Subclinical Hypothyroidism (SCH), Prolactin (PRL), Thyroid stimulating hormone (TSH)

Serial No.	Author's Name	Study Design	Study Summary	Treatment and Study Outcome
1	Olive KE, et al. [5]	Case report	A 45-year-old female presented with Carpal tunnel syndrome and mildobesity. Her thyroxine and prolactin level were 64 (nmol/L) and 187 (Microgram/L)	Levothyroxine sodium 0.1 mg was started. After nine weeks of therapy, the patient was euthyroid and had normal prolactin Levels. After one year of follow-up the patient had normal prolactin levels.
2	Meier C, et al. [6]	Double blinded placebo controlled study	They investigated the effect of levothyroxine treatment on serum prolactin (PRL) levels in women with subclinical hypothyroidism.	Based on the study, they demonstrated that in subclinical hypothyroidism (SCH), prolactin regulation is altered and levothyroxine treatment restores prolactin concentration.
3	Hekimosy Z, et al. [7]	Observational study	They did an observational study to determine the prevalence of hyperprolactinemia in patients with newly diagnosed subclinical and overt hypothyroidism and investigated the change in PRL levels with levothyroxine treatment.	Prolactin level elevation was found in 36% of patients with overt hypothyroidism and in 22% of patients with subclinical hypothyroidism. Prolactin levels decreased to normal in all patients after thyroid functions normalized with levothyroxine treatment. In the hypothyroid patients (overt and subclinical) a positive correlation was found between thyroid stimulating hormone (TSH) and prolactin levels.
4	Tolino A, et al. [8]	Observational study	They studied a correlation between SCH and hyperprolactinemia and sterility.	The results of 25 women aged 22 to 26 years showed a picture of SCH in seven patients (28%), and three patients (12%) had galactorrhea. The increase of volume of thyroid gland was constant in all women with SCH. Authors point out correlation between subclinical hypothyroidism-hyperprolactinemia and sterilty.
5	Empokpae MA, et al. [9]	Cross-sectional prospective study	They studied infertile Nigerian women with hyperprolactinemia with subclinical hypothyroidism.	In conclusion, they explained that SCH was observed in women with hyperprolactinaemia and the ratio of proportions between hyperprolactinemia and hypothyroidism was 7:1; in simple words, in every seven women with hyperprolactinemia, one had hypothyroidism.
6	Atis G, et al. [10]	Cross-sectional study	They did the study to investigate sexual dysfunction in patients with clinical hypothyroidism and SCH.	Female sexual dysfunction was diagnosed in 56% of women with clinical hypothyroidism, 54.6% in women with SCH. The mean prolactin levels were significantly higher in clinical hypothyroidism (30.56), SCH (22.56) as compared to control healthy population (13.03) (ng/ml).
7	Sharma LK, et al. [11]	Cross-sectional prospective study	They evaluated individuals who were either euthyroid or had subclinical or overt hypothyroidism and their correlation with TSH and prolactin.	Significant positive correlation between TSH and prolactin was noted in SCH and primary hypothyroidism.
8	Bahar A, et al. [12]	Cross-sectional Study	They did a cross-sectional study and assessed the prolactin levels of subclinical hypothyroid patients.	They found the prevalence of hyperprolactinemia in SCH was 20.4% (98 patients, 91 females; seven males) and hyperprolactinemia with galactorrhea in 2.6% patients. They concluded that prevalence of hyperprolactinemia in SCH is notable and this disorder is more common in females with subclinical hypothyroidism than in men.

## Conclusions

Hyperprolactinemia with galactorrhea can occur in SCH. In such cases, treatment of SCH with levothyroxine will normalize the prolactin level. Close follow-up of serum prolactin levels should be done before, during, and after treatment in order to avoid unnecessary imaging such as MRI of the brain.
